# Proton beam therapy in Europe: more centres need more research

**DOI:** 10.1038/s41416-018-0329-x

**Published:** 2018-12-11

**Authors:** Marco Durante

**Affiliations:** 1 Biophysics Department, GSI Helmholtz Centre for Heavy Ion Research, Planckstraße 1, Darmstadt, 64291 Germany; 2 Technische Universität Darmstadt, Institute of Condensed Matter Physics, Planckstraße 1, Darmstadt, 64291 Germany

**Keywords:** Radiotherapy, Translational research

Proton beam therapy was applied for the first time in Europe over half a century ago by Börje Larsson, who, in 1957, used the cyclotron of the Gustaf Werner Institute in Uppsala, Sweden, to target a cervical cancer with a high-energy beam of protons.^1^ More centres for proton beam therapy have subsequently been built in Europe since the 1990s. According to the particle therapy co-operative group (PTCOG; available online at www.ptcog.ch), the number of particle therapy facilities in operation in Europe is forecast to increase from the current figure of 21 (as of April 2018) to 45 by 2023.

This rapid growth in the number of proton beam therapy centres, some of which are supported by national governments, others of which are privately funded, is viewed with concern by advocates of evidence-based medicine. A heated debate is still ongoing in the USA, where proton therapy is often perceived as complex and expensive, and centres are being built in the absence of level-1 evidence that proton therapy offers any superiority over conventional X-ray therapy.^2^ Retrospective studies comparing the toxicity of protons and X-rays for prostate cancer failed to find significant advantages in the use of protons.^3^ Moreover, the results of a recent prospective randomised trial in the USA comparing protons to X-ray therapy in locally advanced non-small-cell lung cancer showed no significant differences between the two treatments, in terms of both patient survival and normal tissue complication probability (NTCP).^4^

## Bragg-ing about clinical trials

**Fig. 1:**
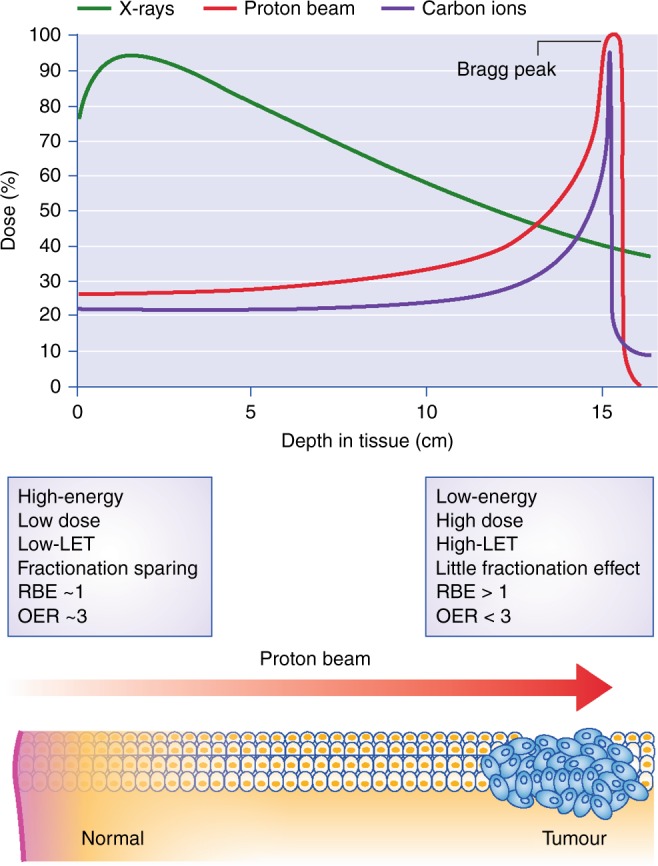
Particle therapy exploits a favourable depth–dose curve. Charged particles deposit most of their initial energy when they slow down, close to the end of the range (Bragg peak) within the tumour target, while X-ray energy decreases exponentially with dose. Particles at high energy (entrance, normal tissue) have linear energy transfer (LET) similar to X-rays, while the LET becomes high when the ions slow down around the Bragg peak. This provides radiobiological advantages such as an increased relative biological effectiveness (RBE) and reduced oxygen enhancement ratio (OER) in the tumour

Numerous phase III clinical trials of charged-particle therapies are currently underway,^5^ the results of which will be important to clarify the real clinical advantages of protons over conventional radiotherapies. The putative advantages of therapy using protons result from their favourable depth-dose distribution.^6^ On interacting with human tissue, charged particles travel predominantly in a straight line, losing only a minimal amount of energy on the way, until they stop at a precise depth that is predetermined by the initial energy given by the accelerator; the amount of energy released at this point reaches its maximum and is known as the Bragg peak (Fig. 1). This approach allows much more of the surrounding normal tissues to be spared than X-ray therapy does (up to 15–30 Gy for brain and head-and-neck tumours). Therefore, comparative trials should detect decreased toxicity if X-ray treatments have a high NTCP, or, an increased local control if the NTCP is kept as high as for X-rays by increasing the total dose to the tumour. Clearly, providing the same tumour dose in locations where toxicity is low will not detect any advantages. The argument of reduced toxicity is used to support the use of protons in paediatric patients, in whom there is indeed evidence of a positive outcome for different later-occurring morbidities.^7^

Useful comparative trials should be based on careful patient selection. The three new proton beam therapy centres in the Netherlands will use an NTCP model to select patients who are expected to benefit significantly from proton therapy.^8^ This is an interesting approach, which is also under consideration in other countries for treatment reimbursement. While a dosimetric advantage is almost always evident using protons, it has been shown that this dose reduction does not always lead to significant decrease in toxicity.^5^

While the model-based approach^8^ seems to be the optimal solution for the selection of patients who will benefit from protons, the problem is that the uncertainties on the NTCP model are too high. More research is needed to develop and test these models. Modern clinical trials in radiotherapy are often based on solid biological hypotheses, and stratify the patients on the basis of molecular biomarkers.^9^ For clinical trials of proton therapy, the selection of patients and trial design based on radiobiology can be essential, given the special radiobiological properties of charged particles compared to X-rays. Radioresistant, hypoxic tumours should preferably be treated with heavy ions. The ability to spare normal tissue might be an advantage in trials that combine immunotherapy with radiotherapy, as higher numbers of naive immune cells will survive treatment with protons than with X-rays.^5^

## Focussed research

In addition to the radiobiological studies mentioned above, more medical physics research is also needed to tackle the problem of range uncertainty, which currently makes proton therapy less robust than conventional radiotherapy, and makes the treatment of moving targets problematic.^6^ The full potential of the Bragg peak has still not been exploited. If the accuracy of proton therapy is increased, the Bragg peak could be viewed as a non-invasive scalpel and potentially used to treat diseases other than cancer, in cranial and extra-cranial targets such as the heart. Stereotactic body radiation therapy, where radiotherapy is administered from different directions, has been recently applied to the treatment of ventricular tachycardia and is considered a possible non-invasive alternative to catheter ablation. Preclinical studies in a swine model^10^ have demonstrated that charged particles are potentially superior to X-rays in the treatment of heart diseases, which would greatly increase the number of patients potentially benefiting from particle therapy.

Alongside the surge in proton beam therapy centres forecast by the PTCOG, are there facilities to perform research in particle therapy in Europe? A few nuclear physics accelerators (such as the GSI/FAIR in Germany; KVI in the Netherlands; and GANIL in France) have active programmes in the field. These large centres should concentrate on new, high-risk–high-gain breakthrough research projects, while also exploiting their abilities to accelerate different ions. More preclinical research in radiobiology and medical physics should be performed in dedicated experimental rooms at the clinical centres. A few of the new centres, such as the APSS proton therapy centre in Trento, Italy, and the PTC centre in Dresden, Germany, have dedicated vaults for research. The new facilities in the UK are planning intense research programs, with dedicated vaults and important collaborations with cancer research centres. These rooms can provide beams for applications beyond therapy, such as testing space radiation shielding and microelectronics damage for use in spacecraft and satellites. Even if beamtime is limited by the clinical activities, it is expected that, with more experimental rooms, research will rapidly grow in the coming years, leading to important innovations and benefits in particle therapy. The contribution of the UK researchers in the field will certainly be of utmost importance.
